# SPP1^+^ macrophages in tumor immunosuppression: mechanisms and therapeutic implications

**DOI:** 10.3389/fimmu.2025.1711015

**Published:** 2025-12-05

**Authors:** Juanjuan Wang, Ya Wang, Yuqing Liu, Rongcun Yang

**Affiliations:** 1 Department of Immunology, Nankai University School of Medicine, Nankai University, Tianjin, China; 2 College of Biotechnology and Food Science, Tianjin University of Commerce, Tianjin, China; 3 State Key Laboratory of Medicinal Chemical Biology, Nankai University, Tianjin, China; 4 Translational Medicine Institute, Affiliated Tianjin Union Medical Center of Nankai University, Nankai University, Tianjin, China

**Keywords:** SPP1+ macrophages, T cell exhaustion, physical barrier, osteopontin, cancer therapy

## Abstract

Secreted phosphoprotein 1 (SPP1^+^) macrophages are a recurrent and functionally critical immune cell subset across multiple cancer types. They drive adverse clinical outcomes by promoting immunosuppression, tumor invasion, metastasis, and therapy resistance. Given their prevalence and pivotal role, SPP1^+^ macrophages have become a major focus in cancer immunology and a promising target for therapeutic development. SPP1^+^ macrophages have been identified in a wide range of human malignancies through single-cell RNA sequencing and spatial transcriptomics studies. Their differentiation and maintenance are strongly influenced by reciprocal cellular interactions and hypoxic conditions within the tumor microenvironment (TME). Within the tumor microenvironment (TME), SPP1^+^ macrophages promote tumor progression by interacting with cancer-associated fibroblasts (CAFs) and helping to form a physical barrier that restricts immune cell infiltration into the tumor core. Specifically, they impair the recruitment of CD8^+^ T cells and promote T cell exhaustion (TEX). In this review, we focus on recent advances in understanding the differentiation of SPP1 macrophages in hypoxic tumor microenvironment and the role of SPP1^+^ macrophages in immunosuppression and their therapeutic implications in cancer. Targeting this subset of macrophages has emerged as a highly promising therapeutic strategy, with several approaches demonstrating encouraging results in preclinical models.

## 1 Introduction

Tumor-associated macrophages (TAMs) are a critical component of the tumor microenvironment (TME) and play multifaceted, often context-dependent roles in cancer progression. Recent single-cell RNA sequencing studies have identified seven major subsets of TAMs, including interferon-mediated tumor-associated macrophages (IFN-TAMs), immunoregulatory TAMs (Reg-TAMs), inflammatory TAM (Inflam-TAMs), lipid-associated- TAM (LA-TAMs), angiogenic TAMs (Angio-TAMs), resident-tissue macrophages like (RTM-TAMs), and proliferating TAM (Prolif-TAMs). Additionally, TAMs can be categorized into eight functional subtypes based on specific gene signatures, including SPP1^+^ TAMs, FOLR2^+^ TAMs, TIE2^+^ TAMs, TREM2^+^ TAMs, MARCO^+^ TAMs, FCN1^+^ TAMs, C1QC^+^ TAMs, and ISG15^+^ TAMs (1). Other subsets have been further annotated with functions such as immunosuppression, lipid metabolism, scavenging, antigen presentation, glycolysis, angiogenesis, hypoxia response, and promotion of tumor cell invasion across various cancer types (2). Among these, SPP1^+^ macrophages have emerged as a functionally distinct and clinically significant subpopulation in multiple cancers (3, 4). These cells are characterized by high expression of the gene SPP1, which encodes the protein osteopontin (OPN). Elevated SPP1 expression is associated with poor prognosis in tumors such as esophageal, colorectal, lung, and ovarian cancers, as well as glioma (5). Consequently, their presence often correlates with unfavorable clinical outcomes, highlighting their potential as targets for novel diagnostic and therapeutic strategies.

SPP1^+^ macrophages originate from two primary sources, tissue-resident macrophages already present prior to tumor development (6), and monocyte-derived macrophages recruited from the circulation via chemotactic signals such as CCL2 (6). Within the TME, factors such as hypoxia, and interaction with tumor cells, fibroblasts and other immune cells polarize these cells toward an SPP1-expressing phenotype. They are commonly localized in hypoxic regions and invasive fronts of tumors (7, 8), and are also abundant at metastatic sites (9). A critical insight from advanced single-cell RNA sequencing and spatial transcriptomics is that SPP1^+^ TAMs are also frequently enriched at the tumor–stroma interface, particularly at the invasive margin (7, 10, 11). This strategic positioning enables them to directly facilitate cancer cell invasion into adjacent normal tissues and suppress antitumor immunity at the tumor frontier. Notably, these SPP1^+^ TAMs display a unique molecular signature that transcends conventional polarization paradigms while contributing significantly to immunosuppression, metastasis, and therapy resistance (9, 12, 13). They induce T cell exhaustion (TEX), promote the formation of immune excluded niche and imped the infiltration of CD8 cells (14). Additionally, they promote angiogenesis via secretion of factors including VEGF, thereby supporting tumor nutrient and oxygen supply (15). The SPP1 protein also enhances cell adhesion, migration, and extracellular matrix (ECM) remodeling, further facilitating local invasion and metastatic dissemination. Overall, SPP1^+^ macrophages play a central role in shaping an immunosuppressive TME and driving cancer progression in malignancies such as ovarian cancer, hepatocellular carcinoma, and head and neck squamous cell carcinoma. Thus, SPP1^+^ TAMs constitute a clinically relevant subset that underscores the complexity of macrophage functions in cancer (16–18). We will here discuss recent advances in understanding the differentiation of SPP1 macrophages in hypoxic tumor environment and the role of SPP1^+^ macrophages in immunosuppression and their therapeutic implications in cancer.

## 2 SPP1 macrophages in tumor tissues

SPP1^+^ macrophages have been identified in a variety of human cancers through single-cell RNA sequencing and spatial transcriptomics studies such as colorectal cancer (CRC) (10, 16, 19), lung cancer (LC) (12, 20), breast cancer (BC) (21, 22), non-small cell lung cancer (NSCLC) (20, 23), hepatocellular carcinoma (HCC) (18), ovarian cancer (24), head and neck squamous cell carcinoma (HNSCC) (25) and anaplastic thyroid cancer (ATC) (26) & pancreatic ductal adenocarcinoma (PDAC) (27, 28).

**Figure 1:**
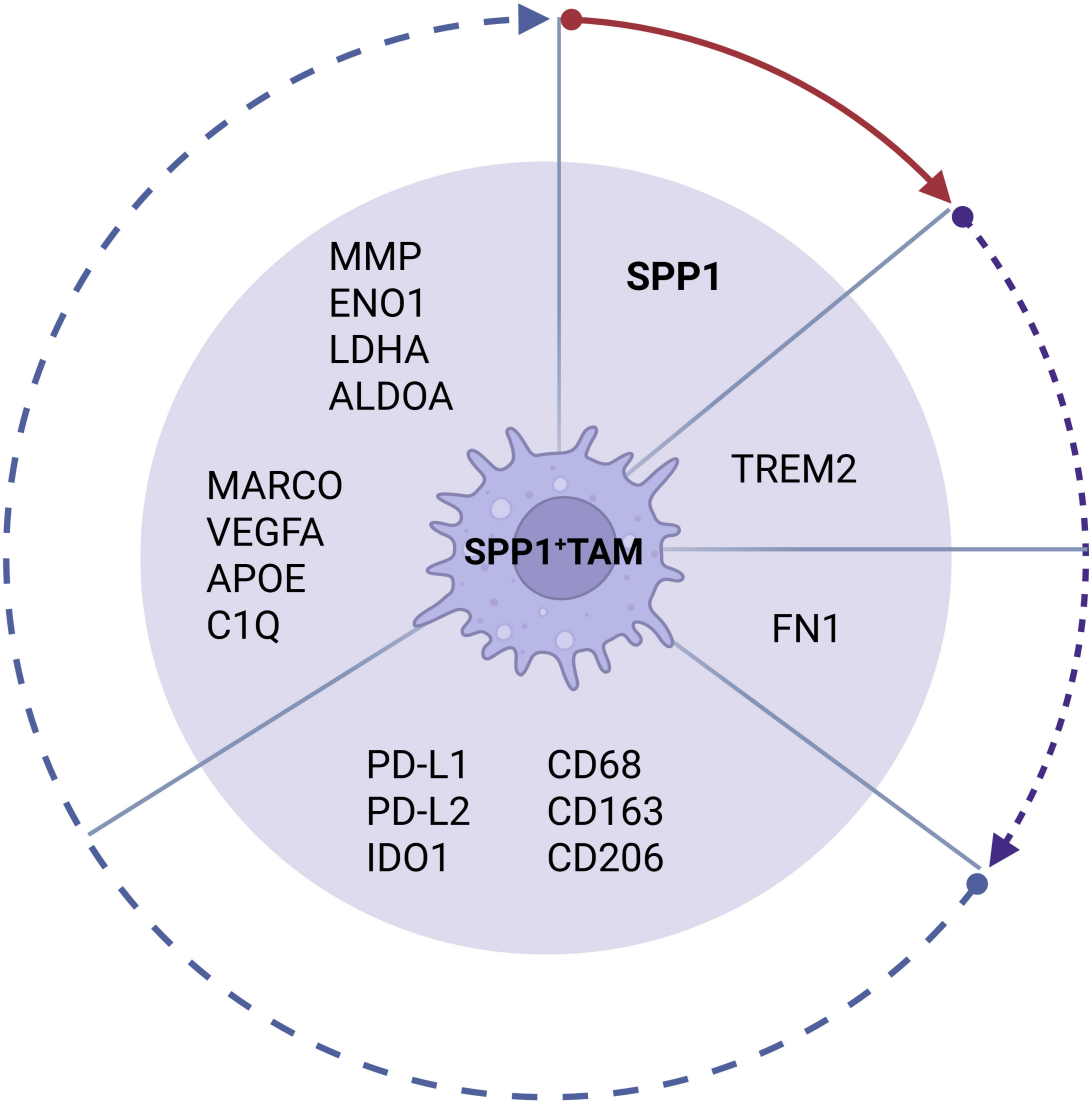
Gene signature profile of SPP1^+^ tumor-associated macrophage (SPP1^+^ TAM) subpopulation in solid tumor tissues. Solid line, SPP1 gene is expressed in SPP1^+^ TAMs in all solid tumor tissues. Closely dotted line, genes TREM2 and FN1 are expressed in SPP1^+^ TAMs in most solid tumor tissues. Dotted line, genes PD-L1, PD-L2, IDO1, CD68, CD163 and CD206, and MARCO, VEGFA, APOE, C1Q, MMP, ENO1, LDHA and ALDPA are only expressed in SPP1^+^ TAMs in some solid tumor tissues. SPP1, secreted phosphoprotein 1; TAM, tumor-associated macrophage; TREM, TREM2, triggering receptor expressed on myeloid cell-2; FN1, fibronectin 1; PD, programmed cell death protein; MARCO, macrophage receptor with collagenous structure; VEGFA, vascular endothelial growth factor A; APOE, apolipoprotein E; C1Q, complement component 1q; MMP, Matrix metalloproteinase; ENO1, enolase 1; LDHA, lactate dehydrogenase; ALDPA, adrenoleukodystrophy protein.

SPP1^+^ macrophages are identified by specific expression of a core gene SPP1. However, SPP1 TAM subpopulation also often expresses the following genes such as *IDO*, *Mrc1(CD206)*, *PD-L1*, *PD-L2*, *CD68*, *CD163* and CD206 (29, 30). Single cell transcriptomics and spatial transcriptomics studies also identified other genes in this subpopulations in different tumors such as *TREM2* and *FN1* in breast cancer (31–33); *MARCO* and *VEGFA* in colorectal cancer (CRC) (34, 35); *ENO1*, *LDHA*, *ALDOA*, and *TPI1* in non-small cell lung cancer (NSCLC); APOE in pancreatic ductal adenocarcinoma (PDAC) (36); metalloproteinases (MMP9, MMP12, and MMP7) in hepatocellular carcinoma (37); *TREM2, C1QC, C1QB, C1QA, SPP1, and APOE* in esophageal squamous cell carcinoma (38) (**Figure 1**). Notably, since some genes related to tissue remodeling and wound healing also appear in embryo-derived tissue-resident macrophages (TRMs) (39–41), SPP1 TAMs might be derived from embryo-derived TRMs (6, 42). However, these SPP1 macrophages are also derived from monocytes (6, 34, 43, 44).

## 3 Differentiation of SPP1 macrophages in tumor microenvironments

Differentiation of SPP1 macrophages in tumor microenvironments depends on hypoxia. SPP1 expression was higher in hypoxia-high macrophages in pan-cancer (45). The expression of SLC2A1, a hypoxia-related molecule promoted the differentiation of SPP1 macrophages in liver metastasis (46). Genetic SPP1 deficiency in macrophages delays hypoxic adaptive tumor growth and enhances the tumor response to anti-programmed cell death-1 (anti-PD-1) therapy (47). Thus, hypoxia critically drives the differentiation and functional polarization of macrophages toward an SPP1^+^ phenotype.

### 3.1 Cellular interactions promote differentiation of SPP1 macrophages in hypoxic tumor microenvironment

**Figure 2:**
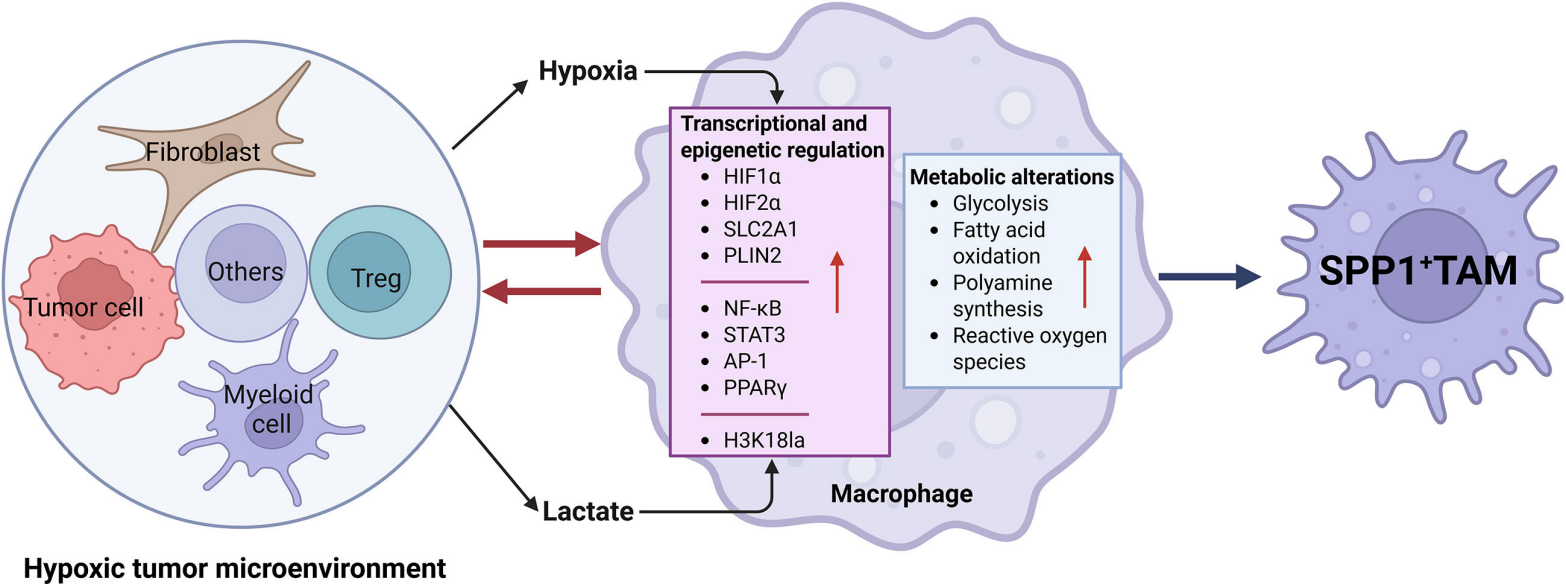
Differentiation of SPP1 macrophages in tumor microenvironment. Macrophages that infiltrate tumor tissues can differentiate into SPP1^+^ macrophages under hypoxic conditions, through interactions with tumor cells, fibroblasts, regulatory T cells, and myeloid-derived suppressor cells. SPP1, secreted phosphoprotein 1; TAM, tumor associated macrophages; Treg, regulatory T cells; HIF, hypoxia-inducible factor; NF-κB, nuclear factor-kappa B; STAT3, signal transducer and activator of transcription 3; AP-1, Activating protein 1.

The generation and maintenance of TAMs are profoundly shaped by reciprocal interactions among tumor cells, stromal components, and other immune cells. Within the intricate tumor ecosystem, the differentiation of macrophages into a specific SPP1-expressing phenotype is not an autonomous process. Rather, it is meticulously orchestrated through a network of bidirectional communications with various cell types. Indeed, recent studies indicate that the hypoxic tumor microenvironment fosters an interplay among its cellular constituents—including cancer cells, cancer-associated fibroblasts, Tregs, MDSCs, and macrophages—that promotes SPP1 macrophage differentiation (**Figure 2**).

#### 3.1.1 Interaction with cancer cells

Tumor cells secrete a wide array of cytokines and chemokines such as CCL2, CSF-1 (M-CSF), IL-6, IL-10, and TGF-β that directly polarize macrophages. Additionally, tumor cells frequently consume substantial amounts of glucose, resulting in a hypoxic and nutrient-depleted microenvironment characterized by low levels of arginine and tryptophan. In triple-negative BC (TNBC), SPP1^+^ macrophage and cancer cell interactions driven by hypoxia signaling correlate with poor prognosis (48). The long-chain fatty acids in a hypoxia-responsive gene SLC27A2-deficient hepatocellular carcinoma (HCC) were taken up by tumor-associated macrophages (TAMs), activating PPAR-γ transcriptional activity and promoting the enrichment of SPP1^+^ TAMs in hepatocellular carcinoma (49).

#### 3.1.2 Interaction with cancer associated fibroblasts

CAFs play a key role in recruiting and instructing macrophages through the secretion of factors such as CCL2, CSF-1, and CXCL12, which promote monocyte recruitment and differentiation into TAMs. CAFs and TAMs engage in a cooperative interplay to remodel the tumor stroma. CAFs deposit dense collagen matrices, while macrophages secrete SPP1, a matricellular protein that modulates cell adhesion, migration, and signaling (10, 50). Immunofluorescence staining confirmed the co-localization of Hypoxia-activated PLAU + fibroblast (iCAF_PLAU) with SPP1 TAM and SPP1 TAM with CD8 T cells in anorectal malignant melanoma (51). iCAF_PLAU secretes CCL2, which binds to CCR1 on SPP1 ^+^ macrophages (TAM_SPP1) cells, leading to the activation of NFKBIA in TAM_SPP1 and subsequent upregulation of IL6, which may be linked to the exhaustion process of CD8 + T cells (51). Hypoxic microenvironment reprogramed CAFs into ERS-CAF subtype, which can regulates SPP1macrophage to aggravate chordoma progression via the IER2/GMFG/ITGB1 axis in chordoma (52). Multiplex immunofluorescence confirmed the enrichment of SPP1^+^CD68^+^TAM and HIF1A^+^SMA^+^CAF in hypoxic triple-negative breast cancer (TNBC) regions (53).

#### 3.1.3 Interaction with immune cells

The crosstalk between macrophages and T cells is bidirectional and reinforces the immunosuppressive niche. The suppression of T cells leads to a deficiency in activating signals such as IFN-γ, thereby permitting macrophages to adopt an alternative, pro-tumoral (M2-like/SPP1^+^) activation state under the influence of tumor-derived factors.

Interaction with other myeloid cells. Tumor-associated neutrophils (TANs) contribute to macrophage polarization by releasing cytokines such as CCL17 and oncostatin M, which promote an M2-like, pro-tumoral phenotype. Furthermore, the immunosuppressive environment can induce dendritic cell (DC) dysfunction. Impaired DC activity reduces the activation of anti-tumor T cells, indirectly facilitating the persistence and polarization of SPP1^+^ TAMs.

#### 3.1.4 Interaction with other cells in tumor microenvironments

PLXDC1^+^ TPSCs, which exhibited cancer-associated myofibroblasts (myCAFs) phenotype linked to poor prognosis in PDAC. Notably, PLXDC1^+^TPSCs located near aggressive LRRC15^+^ myCAFs and SPP1^+^ macrophages could form a desmoplastic and immunosuppressive niche around the tumor boundary, promoting CD8 T cell exhaustion in pancreatic ductal adenoma (28).

### 3.2 Mechanisms of SPP1 macrophage differentiation

The differentiation of SPP1 macrophages is mediated through diverse mechanisms such as epigenetic, signaling, transcriptional, and metabolic pathways (5, 9, 54–57).

#### 3.2.1 Metabolic alterations

TAMs undergo metabolic reprogramming influenced by the TME. Key metabolic pathways involving glucose, lipids, and amino acids are often manipulated (58). Hypoxia induces SPP1 expression in macrophages (45, 46, 59) primarily through HIF-1α, which promotes a shift toward glycolytic metabolism and upregulates SPP1. SPP1^+^ macrophages exhibit enhanced glycolytic activity, supporting their survival under hypoxia and facilitating the production of pro-inflammatory cytokines such as TNF-α and IL-1β. SPP1^+^ TAM subsets also often display increased fatty acid oxidation (FAO), which reinforces an immunosuppressive phenotype and further promotes SPP1 expression (23, 60). Peroxisome proliferator-activated receptor gamma (PPARγ), a key regulator of lipid metabolism, contributes to the differentiation of SPP1^+^ macrophages (61) by enhancing the expression of genes involved in lipid handling and immune suppression. Competition for arginine in the TME leads to its metabolism by arginase-1 (Arg1), which is highly active in SPP1^+^ macrophages. This promotes polyamine synthesis and supports tumor progression and immunosuppression. Additionally, indoleamine 2,3-dioxygenase (IDO) catabolizes tryptophan, contributing to an immunosuppressive TME (62). Although not directly linked to SPP1 in the cited literature, IDO activity is commonly associated with M2-like macrophage polarization, which may include SPP1^+^ subsets.

#### 3.2.2 Transcriptional and epigenetic regulation

Under low oxygen conditions, the transcription factors HIF-1α and HIF-2α are stabilized and directly upregulate the expression of multiple genes in macrophages, including SPP1, VEGF, and key enzymes involved in anaerobic glycolysis (24, 63). In SPP1^+^ macrophages, NF-κB activation drives the expression of pro-inflammatory cytokines such as TNF-α and IL-1β (64). SPP1 can engage receptors like CD44, activating NF-κB signaling and establishing a feed-forward loop that sustains macrophage polarization and promotes tumor progression. STAT3 is implicated in the epigenetic regulation of SPP1 expression. In aggressive cancers (e.g., pancreatic and thyroid cancer), lactate-induced histone H3 lysine 18 lactylation (H3K18la), mediated by VSIG4^+^ TAMs activates STAT3, which binds to the SPP1 promoter and enhances its transcription (26). Elevated lactate levels in the TME promote histone lactylation, enabling STAT3 to epigenetically upregulate SPP1. Hypoxia stabilizes HIF-1α, which indirectly supports SPP1 expression by promoting glycolytic metabolism and lactate production (24). Notably, emerging evidence highlights a link between cancer cell metabolism and epigenetic reprogramming of TAMs via histone lactylation, which may underlie sustained protumoral polarization of TAMs (5, 18, 65).

## 4 SPP1^+^ macrophages in tumor immunosuppression

These SPP1 macrophages contribute to immunosuppression through various mechanisms, including fostering the formation of an immunosuppressive niche, inducing TEX, inhibiting CD8^+^ T cell infiltration, and enhancing the recruitment of other immunosuppressive cell types.

SPP1 macrophages typically different from other macrophages and exert their specific functions through interaction of SPP1 with their receptors on the targeting cells. SPP1 functions as a bridging molecule between cells and the extracellular matrix by binding to various integrins, including αvβ1, αvβ3, αvβ5, α5β1, α8β1, and α4β1as well as to CD44 splice variants such as CD44v6 and CD44v7 (66). Receptor signaling often involves cooperation between integrins and CD44. Proteolytic cleavage by enzymes such as thrombin can generate osteopontin fragments with enhanced bioactivity, revealing new binding sites that interact with higher affinity to specific integrins like α4β1 and α9β1.

### 4.1 SPP1^+^ macrophages drive tumor progression via crosstalk with CAFs

CAF populations found in primary and metastatic cancers have been widely studied and are implicated in tumor initiation, progression and metastasis (67, 68). CAFs undergo epigenetic changes to produce secreted factors, exosomes and metabolites that influence tumor angiogenesis, immunology and metabolism. Single-cell and spatial analysis have shown that SPP1 TAMs are positively associated with FAP^+^ CAFs (69).

A number of mesenchymal cell biomarkers have been used to identify CAFs, including but not limited to αSMA, fibroblast-specific protein 1 (FSP1, also known as S100A4), fibroblast activation protein (FAP), platelet-derived growth factor receptor-α (PDGFRα), PDGFRβ, desmin, discoidin domain-containing receptor 2 (DDR2) and vimentin (69). More recently, the advent of scRNA-seq has enabled a deeper understanding of CAF heterogeneity across a wide range of tumor types, leading to the identification of a variety of biomarker genes defining different potential subpopulations of CAFs such as αSMA, FAP (encoded by *FAP*), decorin (encoded by *DCN*) and/or podoplanin (encoded by *PDPN*) (69).

**Figure 3:**
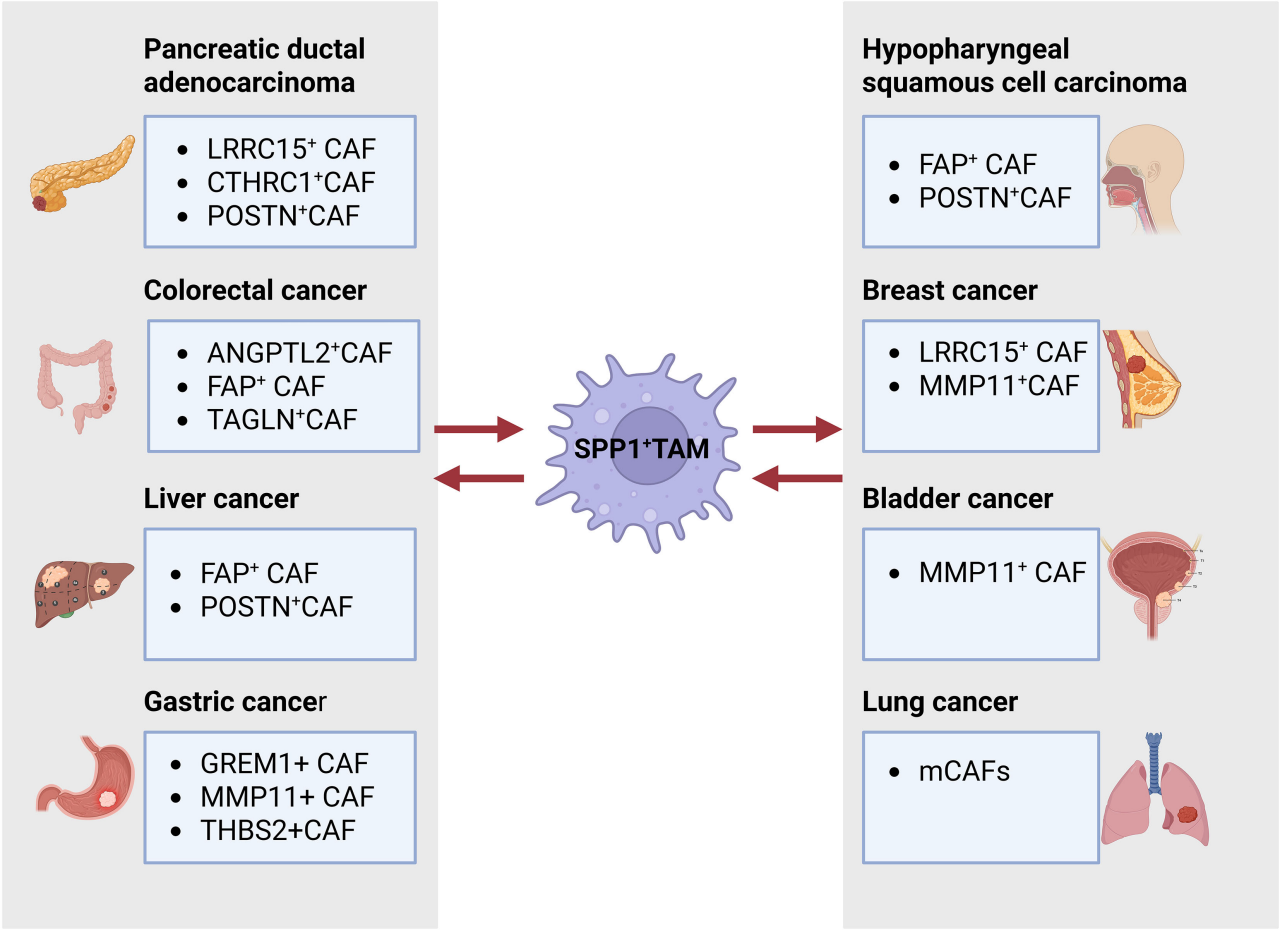
Interaction between SPP1^+^ tumor-associated macrophages (TAMs) and cancer-associated fibroblasts (CAFs) drives tumor progression. Diverse CAF subpopulations identified across various tumor types can engage with SPP1^+^ TAMs within solid tumor tissues to promote this process.

The interaction between SPP1 macrophages and tumor associated fibroflasts exert important functions such as determines the efficacy of immunotherapy (7, 18), metastasis angiogenesis (70–73); immune suppression of cancer (74, 75), CD8 T cell exhaustion (28). Interactions between SPP1+ macrophages and CAFs were identified across multiple tumor types (**Figure 3**) such as SPP1 macrophages and FAP^+^CAFs (10, 76, 77), ANGPTL2^+^CAFs (73), TAGLN^+^ CAFs (78) in colorectal cancer; POSTN^+^, POSTN+ and FAP+CAFs in liver cancer (18); LRRC15^+^myCAFs (18), CTHRC1^+^CAFs (36), POSTN^+^ CAFs in PDAC; FAP^+^ CAFs (76, 77, 79); GREM1^+^ CAFs (80), MMP11^+^ mCAFs (70) in gastric cancer; MMP11^+^ mCAF (70–72) in Bladder Cancer; LRRC15^+^ myCAFs, Vascular CAFs, matrix CAFs, cycling (proliferative) CAFs and developmental CAFs (81); MMP11^+^ mCAFs (70) in human breast tumors (82); POSTN^+^ CAFs (74) in non-small cell lung cancer. Notably, the crosstalk between SPP1^+^ macrophages and FAP^+^ CAFs was found to promote tumor progression (**Figure 3**).

### 4.2 SPP1^+^ macrophages foster immunosuppressive niche formation through CAFs

**Figure 4:**
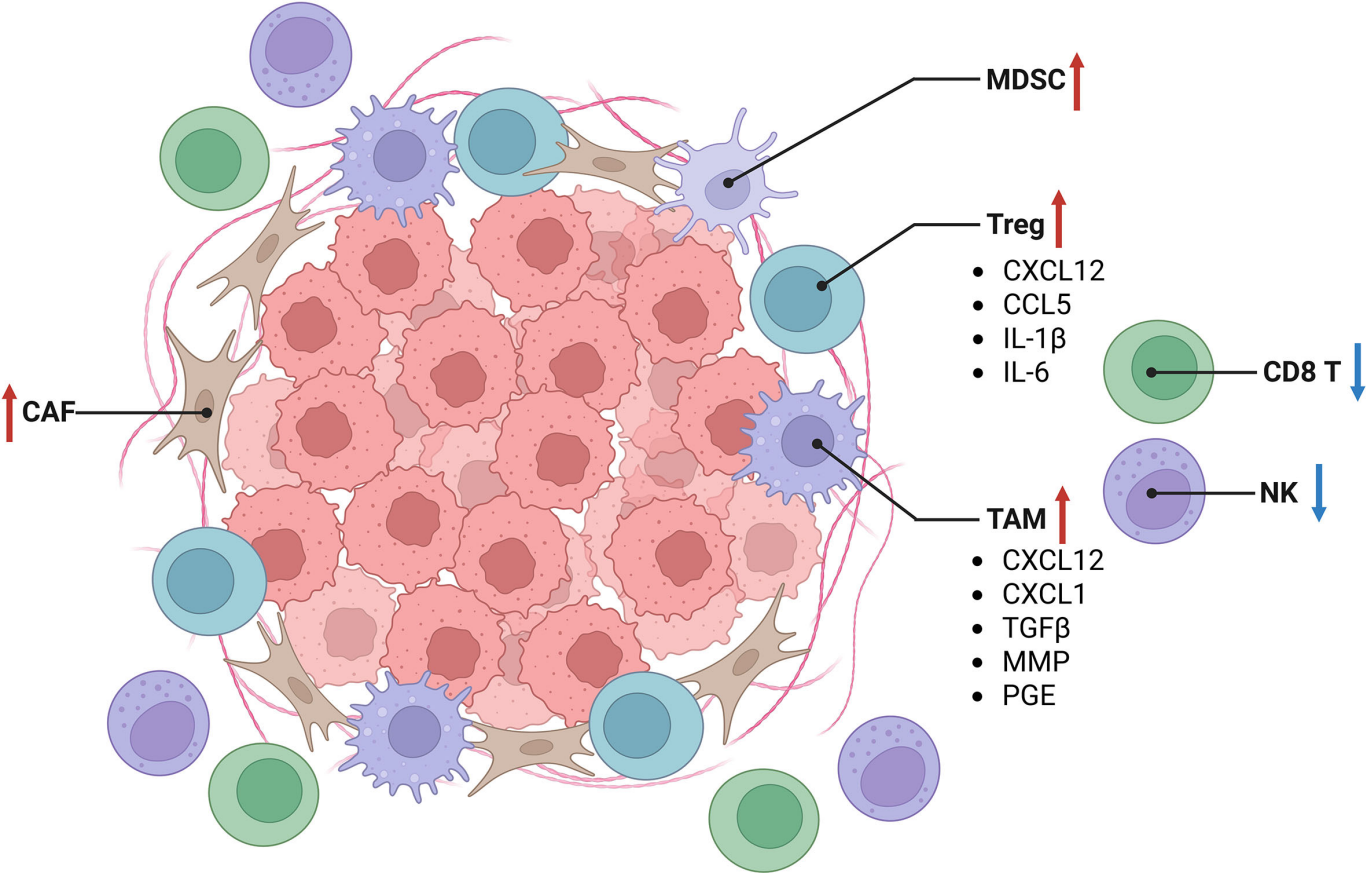
Characteristics of immunosuppressive niche in tumor microenvironment. Tumor-associated fibroblasts and other stromal constituents, including tumor-associated macrophages, can establish a specialized niche within the tumor microenvironment that impairs the cytotoxic function of effector CD8^+^ T lymphocytes and natural killer cells. CAF, cancer associated fibroblast; TAM, tumor associated macrophage; Treg, regulatory T cell; MDSC, myeloid derived suppressor cell; NK, natural killer; CCL, C-C motif chemokine ligand; CXCL, chemokine (C-X-C motif) ligand; TGFβ, transforming growth factor (TGF)-beta; MMP, Matrix metalloproteinase.

CAFs are abundant in most solid malignancies and are established as critical regulators of cancer progression and therapeutic response (56, 83–86). CAFs secrete fibronectin and collagen, contributing to the formation of immunosuppressive niche that not only impede effector T cell infiltration but also promote an immune “desert,” thereby suppressing antitumor immunity (87). To formation of immunosuppressive niche, they interact with various components of TME, including tumor cells, TAMs, and other immune cells, through multiple mechanisms such as direct ligand–receptor interactions, autocrine and paracrine signaling, and ECM remodeling (88, 89). The immunosuppressive niche can influence primary tumor growth, metastasis, therapy resistance, and immunosuppression (90, 91) (**Figure 4**).

CAFs release cytokines, chemokines, immunosuppressive factors, exosomes, and matrix components into the extracellular space, thereby modulating the behavior of surrounding cells (92). Through paracrine signaling mediated by cytokines (e.g., TGF-β, IL-6) and chemokines (e.g., CXCL12, CCL2), CAFs recruit immunosuppressive cells such as M2 macrophages, N2 neutrophils, MDSCs, and Tregs. They also suppress dendritic cell maturation and antigen presentation, promote the differentiation of monocytes into M2-like TAMs and of T cells into Tregs, and impair the effector functions of cytotoxic immune cells including CD8^+^ T cells. TGF-β, abundantly secreted by CAFs, exerts multiple pro-tumor effects within the TME (93). CXCL12, primarily produced by CAFs, binds to CXCR4 or CXCR7 to recruit specific immune cell subsets, fostering an immunosuppressive TME and facilitating tumor progression (94). Additionally, CAFs mediate intercellular communication and metabolic reprogramming via exosomes containing circRNAs, miRNAs, and metabolites. By activating integrin signaling pathways and secreting matrix metalloproteinases, CAFs promote ECM remodeling and increase stromal stiffness, further enhancing physical barriers that limit T cell infiltration (83). Hypoxia resulting from CAF accumulation stimulates the secretion of inhibitory molecules such as VEGF, which disrupt dendritic cell and T cell differentiation and upregulate immune checkpoint molecules including PD-L1 and CTLA-4, thereby promoting immune evasion and therapy resistance (51). High CAF abundance is also associated with reduced responses to anti-PD-1/PD-L1 and anti-CTLA-4 therapies (95).Crosstalk between TAMs and CAFs plays a central role in shaping the immunosuppressive niche. TAMs are a major immune population in solid cancers and significantly contribute to disease progression (86). CAFs critically influence TAM phenotype and function by reprogramming tissue-infiltrating monocytes and modulating their survival and differentiation. This reciprocal CAF–TAM interaction represents a key axis in establishing an immunosuppressive TME through diverse physical and soluble signals (86). Fibroblast-derived colony-stimulating factor 1 (CSF-1) supports macrophage survival and proliferation both under steady-state conditions and in cancer (96). CAFs also secrete various cytokines (e.g., TNFα, IL-1β, IL-6, IL-8, IL-10, IL-33, TGFβ) and chemokines, which are critical for recruiting monocytes and driving their differentiation into immunosuppressive TAMs (97).

SPP1^+^ macrophages are involved in the immunosuppressive niche formation. In tumor tissues, SPP1^+^ macrophages engage in close crosstalk with FAP^+^ CAFs through specific signaling pathways, resulting in the immunosuppressive niche formation (10) (7, 16, 18, 98). The involvement of SPP1^+^ macrophages in this exclusionary barrier has been observed across multiple cancer types, including colorectal cancer (10, 16, 73), hepatocellular carcinoma (7, 18, 98, 99), non-small cell lung cancer (74, 79), gastric carcinoma (100), prostate cancer (76), esophageal squamous cell carcinoma (13), and bladder cancer (70). In colorectal cancer (CRC), interactions between SPP1^+^ macrophages and FAP^+^ fibroblasts, mediated by macrophage-derived factors such as TGFB1 and IL1A/B drive the development of a fibrotic barrier along the tumor margin (10). In metastatic CRC to the liver, transcriptional reprogramming of macrophages and their interaction with fibroblasts support tumor growth within an immunosuppressive metastatic niche (16). In hepatocellular carcinoma (HCC), SPP1^+^ macrophages co-localize with CAFs near the tumor border, forming a “Tumor Immune Barrier” (TIB) (7, 18, 98). Through ligand–receptor interactions such as SPP1 binding to integrins ITGAV, ITGB1, and ITGB5 on CAFs, these macrophages promote extracellular matrix (ECM) remodeling by CAFs. The resulting α-SMA-rich fibrotic regions are associated with resistance to immune checkpoint blockade (ICB) in HCC patients (7). Moreover, DAB2^+^ and SPP1^+^ TAMs enhance the activity of FAP^+^ CAFs via signaling molecules including TGF-β, PDGF, and ADM. These interactions facilitate barrier formation and correlate with poor survival and lack of response to immunotherapy in HCC (98). Additionally, POSTN^+^ CAFs recruit SPP1^+^ macrophages and upregulate SPP1 expression through the IL-6/STAT3 pathway (98). In pancreatic ductal adenocarcinoma (PDAC), PLXDC1^+^ tumor-associated pancreatic stellate cells (TPSCs) are located adjacent to LRRC15^+^ myCAFs and SPP1^+^ macrophages, collectively forming a desmoplastic and immunosuppressive perimeter that promotes CD8^+^ TEX (28). Single-cell transcriptomic data from ICB-treated PDAC patients confirmed that PLXDC1^+^ TPSCs are associated with poor therapeutic outcomes (28). In gastric cancer, crosstalk between CAFs and macrophages modulates the response to ICB in peritoneal metastases, with macrophage-derived MIF contributing to immunosuppression via the MIF–CD74/CXCR4/CD44 axis (100, 101). In non-small cell lung cancer (NSCLC), multiplex staining reveals enriched interactions between SPP1^+^ macrophages and FAP^+^ fibroblasts. NicheNet analysis implicates the VCAN–ITGB1 ligand–receptor axis in this interaction, and spatial transcriptomics confirms its role in establishing an immune-excluded architecture around the tumor core (79).

### 4.3 SPP1 macrophages induce TEX

**Figure 5:**
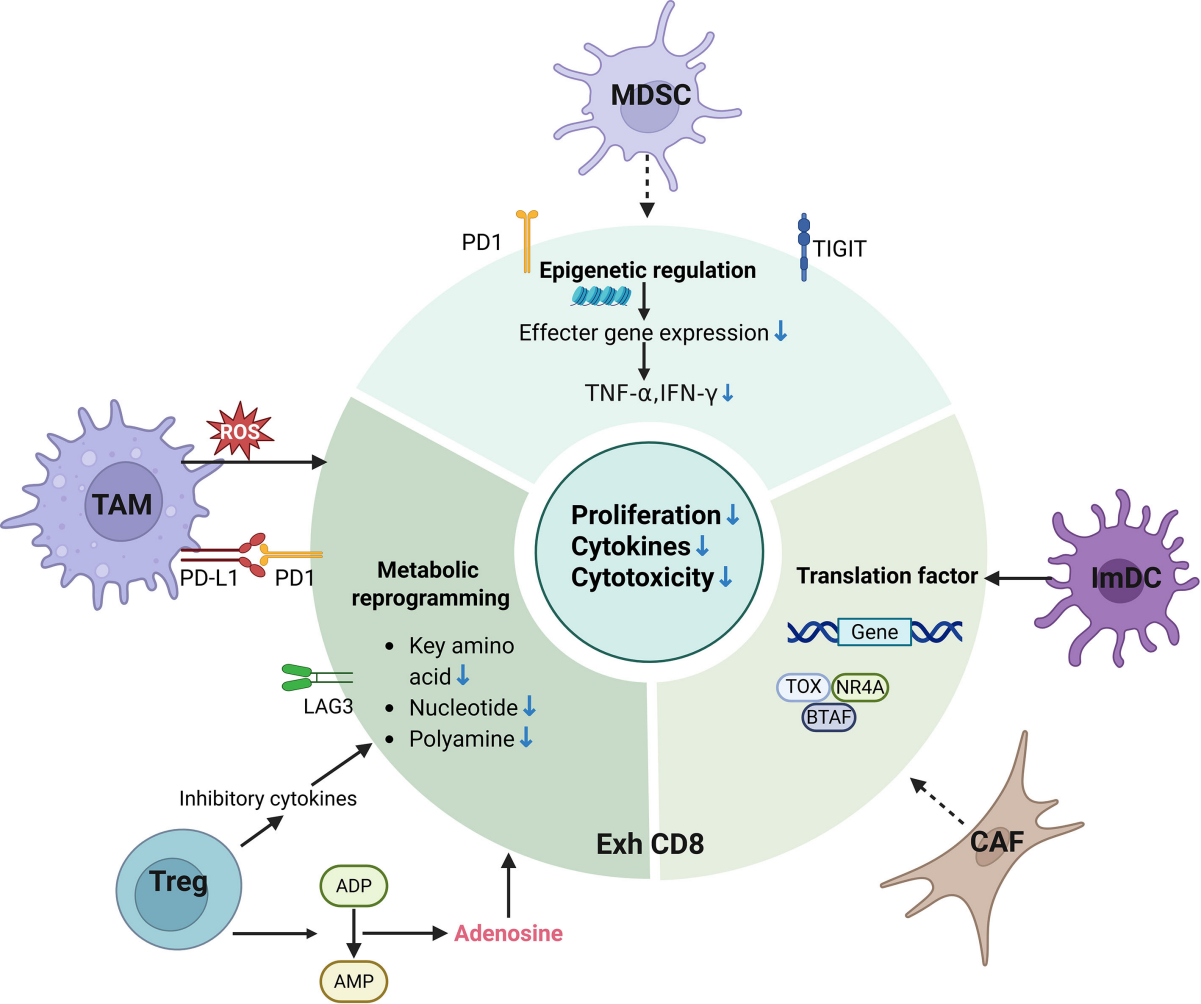
T cell exhaustion in tumor microenvironment. Key immunosuppressive components in the tumor microenvironment, including tumor-associated macrophages, regulatory T cells, myeloid-derived suppressor cells, and cancer-associated fibroblasts (CAFs, are major drivers of T cell exhaustion. TAM, tumor associated macrophage; Treg, regulatory T cell; MDSC, myeloid derived suppressor cell; NK, natural killer; DC, dendritic cell; PD-1, programmed death-1; LAG3, lymphocyte activation gene 3; CTLA-4, Cytotoxic T-lymphocyte antigen-4; TIGIT, T cell immunoreceptor with Ig and ITIM domains; T EXH, T cell exhaustion; ATP, adenosine-5’-triphosphate; AMP, Adenosine monophosphate; ROS, reactive oxygen species; TOX, thymocyte selection-associated high mobility group box; NR4A, nuclear receptor subfamily 4 group A; BATF, Basic leucine zipper transcription factor ATF-like; TNF-α, Tumor necrosis factor-alpha; IFN-γ, interferon –gamma.

CD8^+^ T cells are critical cytotoxic mediators of antitumor immunity. Under some conditions such as TME, they become hyporesponsive, entering a state known as TEX (102). TEX is distinct from cell death; it represents a dysfunctional state in which T cells lose their capacity to kill target cells (103). Exhausted T cells are defined by the expression of inhibitory receptors (e.g., PD-1, TIM-3, LAG-3), loss of effector functions, including reduced production of cytokines such as IFN-γ, TNF-α, and IL-2Altered metabolic profile and diminished proliferative capacity, and a distinct epigenetic and transcriptional signature (104, 105). The TME can drive TEX through multiple factors such as persistent antigen stimulation, upregulation of immune checkpoints, recruitment of immunosuppressive cells, and secretion of immunosuppressive cytokines (93, 103) (**Figure 5**).

#### 4.3.1 Induction of TEX

##### 4.3.1.1 Signaling pathways inducing TEX

Hypoxia within the tumor microenvironment (TME) upregulates HIF-1α, which promotes the differentiation of monocytic myeloid-derived suppressor cells (m-MDSCs) into tumor-associated macrophages (TAMs). These TAMs inhibit T cell activation and effector functions (103). Additionally, IL-10 secreted by MDSCs facilitates the recruitment of CD4^+^ T cells into the TME and their differentiation into regulatory T cells (Tregs) in the presence of TGF-β. The interplay between IL-35 (released by CD177^+^ Tregs) and IL-10 (produced by PD-1^+^ Tregs) further suppresses CD8^+^ T cell cytotoxicity and promotes TEX (103). The transcription factor Osr2 recruits HDAC3 to remodel the epigenetic landscape, repressing the expression of cytotoxic genes and driving CD8^+^ TEX (106). Taurine deficiency in CD8^+^ T cells increases endoplasmic reticulum (ER) stress, leading to ATF4 transcription via the PERK–JAK1–STAT3 pathway. Elevated ATF4 transactivates multiple immune checkpoint genes and accelerates TEX (107). In melanoma mouse models, CD8^+^ T cells deficient in both PD-1 and LAG-3 show improved tumor clearance and prolonged survival compared to single-receptor knockout cells (108). Ablation of β1-adrenergic signaling impedes TEX during chronic infection and enhances effector function when combined with immune checkpoint blockade (ICB) in melanoma models (109). Chronic stimulation induces Blimp-1-mediated repression of PGC-1α-dependent mitochondrial reprogramming, reducing cellular responsiveness to hypoxia. Mitochondrial dysfunction increases reactive oxygen species (ROS) to intolerable levels, promoting an exhausted-like state partly through phosphatase inhibition and subsequent NFAT activation. Reducing T cell-intrinsic ROS and alleviating tumor hypoxia attenuate TEX and synergize with immunotherapy (110). In hepatocellular carcinoma (HCC), promoter hypermethylation silences the E3 ubiquitin ligase Riplet. Loss of Riplet disrupts fatty acid metabolism, driving terminal exhaustion of CD8^+^ T cells (111). HCC-derived exosomes induce neutrophil infiltration and TEX in the livers of DEN/CCl_4_-induced HCC mice, promoting tumor progression (112).

##### 4.3.1.2 Metabolic reprogramming

Different T cell subsets utilize distinct metabolic programs to support their functions. Effector T cells rely on glycolysis to meet high energy demands for proliferation, differentiation, and effector functions. Memory T cells predominantly use fatty acid oxidation (FAO) and oxidative phosphorylation (OXPHOS), while exhausted T cells largely depend on glycolysis (113). Metabolic reprogramming during T cell activation and differentiation plays a critical role in determining T cell fate and immune responses. Cellular metabolism can drive epigenetic changes that regulate T cell differentiation (113). Growing evidence indicates that exhausted T cells display metabolic impairments and altered signaling pathways (114). Under chronic antigen exposure, T cells gradually develop exhaustion, a process closely associated with mitochondrial dysfunction. Strategies targeting mitochondrial metabolism such as inducing PGC-1α expression, reducing ROS, ameliorating hypoxia, enhancing ATP production, and employing mitochondrial transfer can reverse TEX (115, 116).

##### 4.3.1.3 Transcriptional and epigenetic regulation

Several transcriptional regulators are involved in CD8^+^ T cell exhaustion, including Blimp-1, VHL, Eomes, Foxo1, IRF4, BATF, and NFAT (117). Multi-level epigenetic profiling such as transcription factor binding, histone modifications, DNA methylation, and 3D genome architecture has refined our understanding of TEX (114, 118). Studies in human cancers have revealed conserved epigenetic features between murine and human exhausted T cells (119–121). ATAC–seq analysis of exhausted T cells in human basal cell carcinoma identified approximately 4,500 differentially accessible regions (121). A recent integrated reanalysis of over 300 human and mouse ATAC–seq and RNA-seq datasets from CD8^+^ T cells in chronic infection and cancer demonstrated highly similar global chromatin landscapes across conditions and species, despite organism-specific enhancer divergence at certain gene loci (122).

#### 4.3.2 SPP1^+^ macrophages promote TEX

**Figure 6:**
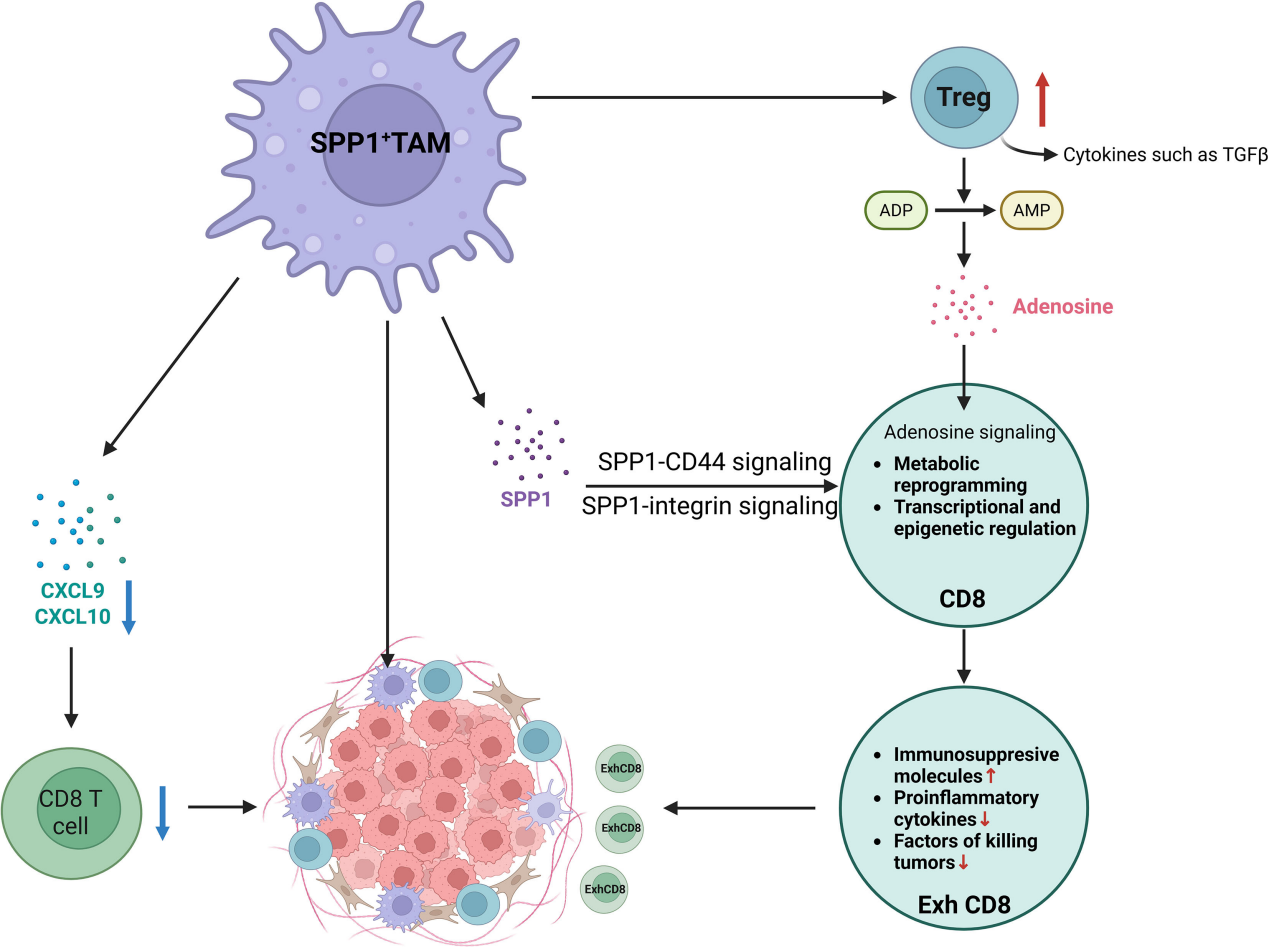
Immunosuppressive functions of SPP1 macrophages in tumor microenvironment. SPP1-expressing macrophages within the tumor microenvironment are established contributors to immunosuppression. Their mechanisms include promoting T cell exhaustion, suppressing the infiltration of cytotoxic CD8+ T cells, and fostering the development of immunosuppressive niches. SPP1, secreted phosphoprotein 1; ATP, Adenosine-5’-triphosphate; AMP, Adenosine monophosphate; Exh, exhaustion; Treg, regulatory T cell; CXCL, C-X-C motif chemokine ligand; TAM, tumor associated macrophage.

SPP1^+^ macrophages promote TEX via CD44 receptors, integrins and immunosuppressive Treg. Additionally, CD73 expressed in SPP1^+^ macrophages also suppress CD8^+^ T cell function through the adenosine–A2AR pathway (**Figure 6**).

1. SPP1^+^ macrophages secrete SPP1, which binds to CD44 receptors on T cells. This interaction initiates a cascade of intracellular signaling events that profoundly alter T cell function. Specifically, binding of SPP1 to CD44 induces a dose-dependent increase in the expression of exhaustion markers such as PD-1, LAG-3, and other inhibitory receptors (123, 124). In ovarian cancer, SPP1^+^ tumor-associated macrophages (TAMs) promote immune suppression and T cell exhaustion (TEX) through the SPP1–CD44 axis, highlighting this pathway as a promising therapeutic target for reprogramming the immunosuppressive tumor microenvironment and improving patient outcomes (125). Similarly, in alpha-fetoprotein-negative hepatocellular carcinoma (AFP-negative HCC), both SPP1^+^ TAMs and CD44 expression on T cells and tumor cells are highly enriched (126). Targeting the SPP1–CD44 axis with anti-SPP1 or anti-CD44 antibodies, either alone or in combination with anti–PD-1, restored T cell function *in vitro* and significantly reduced tumor burden in mouse models (126). High-dimensional multi-omics analysis of patient samples further revealed an elevated frequency of exhausted tumor-reactive CD8^+^ T cells and enhanced interactions between these T cells and SPP1^+^ macrophages, particularly within profibrotic, α-SMA-rich regions of the liver (9). In intrahepatic cholangiocarcinoma (ICC), tumor cells located at the leading edge exhibit high proliferative activity and are closely associated with stromal components, including endothelial cells and POSTN^+^ FAP^+^ fibroblasts. CD8^+^ T cells in these regions display a naïve phenotype, characterized by low cytotoxicity and features of exhaustion, likely due to impaired antigen presentation by local antigen-presenting cells (APCs). Of note, mucosal-associated invariant T (MAIT) cells, which represent the predominant CD8^+^ subset, recruit SPP1^+^ macrophages into the stroma. This recruitment, together with the presence of POSTN^+^ cancer-associated fibroblasts (CAFs) and endothelial cells, forms a distinctive “triad structure” that facilitates tumor growth and ICC progression (75).
2. SPP1 binds several integrin receptors on T cells such as α4β1 and α9β1, activating downstream signaling pathways that form a complex regulatory network influencing T cell behavior (127).
3. SPP1^hi^ TAMs exhibit elevated expression of immunosuppressive genes, including those involved in adenosine signaling such as CD73. Adenosine functions as a potent immunosuppressive metabolite generated through the hydrolysis of extracellular ATP by ectonucleotidases CD39 and CD73 (128). It signals via G-protein-coupled receptors, with the A2A receptor (A2AR) playing a central role in mediating T cell suppression. Activation of A2AR triggers cAMP–PKA signaling, which attenuates TCR activation and restrains T cell proliferation, effector functions, and the production of antitumor cytokines including IL-2, IFN-γ, and TNF-α. The adenosine–A2AR axis further promotes T cell anergy and exhaustion, while also impairing chemokine secretion, thereby compromising T cell recruitment and infiltration into tumor sites (129–131). Notably, in tumors such as gastric cancer (GC), regulatory T cells (Tregs) contribute to this immunosuppressive milieu by converting ATP to adenosine, leading to A2aR-mediated induction of CD8^+^ T cell apoptosis and suppression (128).

### 4.3 SPP1^+^ macrophages inhibit infiltration of CD8^+^ T cells

Our recent study demonstrates that macrophage-derived SPP1 critically impairs CD8^+^ T cell infiltration into the tumor microenvironment (TME), thereby accelerating tumor progression and diminishing the efficacy of immune checkpoint inhibitor (ICI) therapy. We further showed that SPP1 deficiency in macrophages elevates mitochondrial reactive oxygen species (ROS) production, leading to the accumulation of cytosolic double-stranded DNA (dsDNA) fragments. This dsDNA accumulation triggered activation of the cGAS–STING pathway, followed by phosphorylation of STAT1. Enhanced STAT1 signaling upregulated the expression of the chemokines CXCL9 and CXCL10, which facilitated the recruitment of CD8^+^ T cells into the TME.

### 4.4 SPP1^+^ macrophages enhance recruitment of immunosuppressive cells

#### 4.4.1 Tregs

Co-expression and interactions between SPP1+ tumor-associated macrophages (TAMs) and regulatory T cells (Tregs) have been identified within CD44-enriched regions in the TME (132). SPP1^+^ macrophages typically promote pro-tolerogenic and immunosuppressive responses within the TME and inflammatory sites. A key function of these macrophages is to enhance the expansion, stability, and immunosuppressive capacity of Tregs through multiple direct and indirect mechanisms. Specifically, SPP1+ macrophages represent a significant source of TGF-β, which is critical for the differentiation of naïve CD4^+^ T cells into induced Tregs (iTregs) and for sustaining the suppressive function of established Tregs. They also secrete IL-10, which reinforces a tolerogenic milieu and augments Treg activity. Furthermore, these macrophages often express immune checkpoint ligands such as PD-L1. The interaction between PD-L1 and PD-1 on effector T cells suppresses Teff activation, thereby indirectly favoring Treg dominance. Additionally, SPP1^+^ macrophages frequently upregulate arginase 1 (Arg1), leading to depletion of L-arginine, an amino acid essential for T cell function. This metabolic reprogramming creates a selective niche that supports Treg survival while inhibiting anti-tumor effector T cells.

#### 4.4.2 MDSCs. SPP1^+^ macrophages play a key role in recruiting, expanding, and enhancing the immunosuppressive function of MDSCs

They secrete chemokines that directly attract MDSCs to inflammatory sites or the TME. SPP1^+^ macrophages often release CCL2, which binds to CCR2 highly expressed on monocytic MDSCs (M-MDSCs), guiding their migration. They may also produce ligands for CXCR2 (e.g., CXCL1, CXCL2, CXCL5, CXCL8/IL-8), facilitating the recruitment of granulocytic/polymorphonuclear MDSCs (PMN-MDSCs). Furthermore, SPP1 itself can act as a chemoattractant by binding to integrins (such as α4β1, α9β1, αvβ3) and CD44 variants on MDSCs, promoting their adhesion and recruitment. These macrophages secrete pro-inflammatory cytokines including IL-1β, IL-6, and TNF-α, which promote MDSC activation and expansion via STAT3 and other signaling pathways. The cytokine milieu generated by SPP1^+^ macrophages helps maintain MDSCs in an immature, immunosuppressive state, inhibiting their differentiation into antigen-presenting cells such as dendritic cells or macrophages.

## 5 Therapeutic implications and targeting strategies

**Figure 7:**
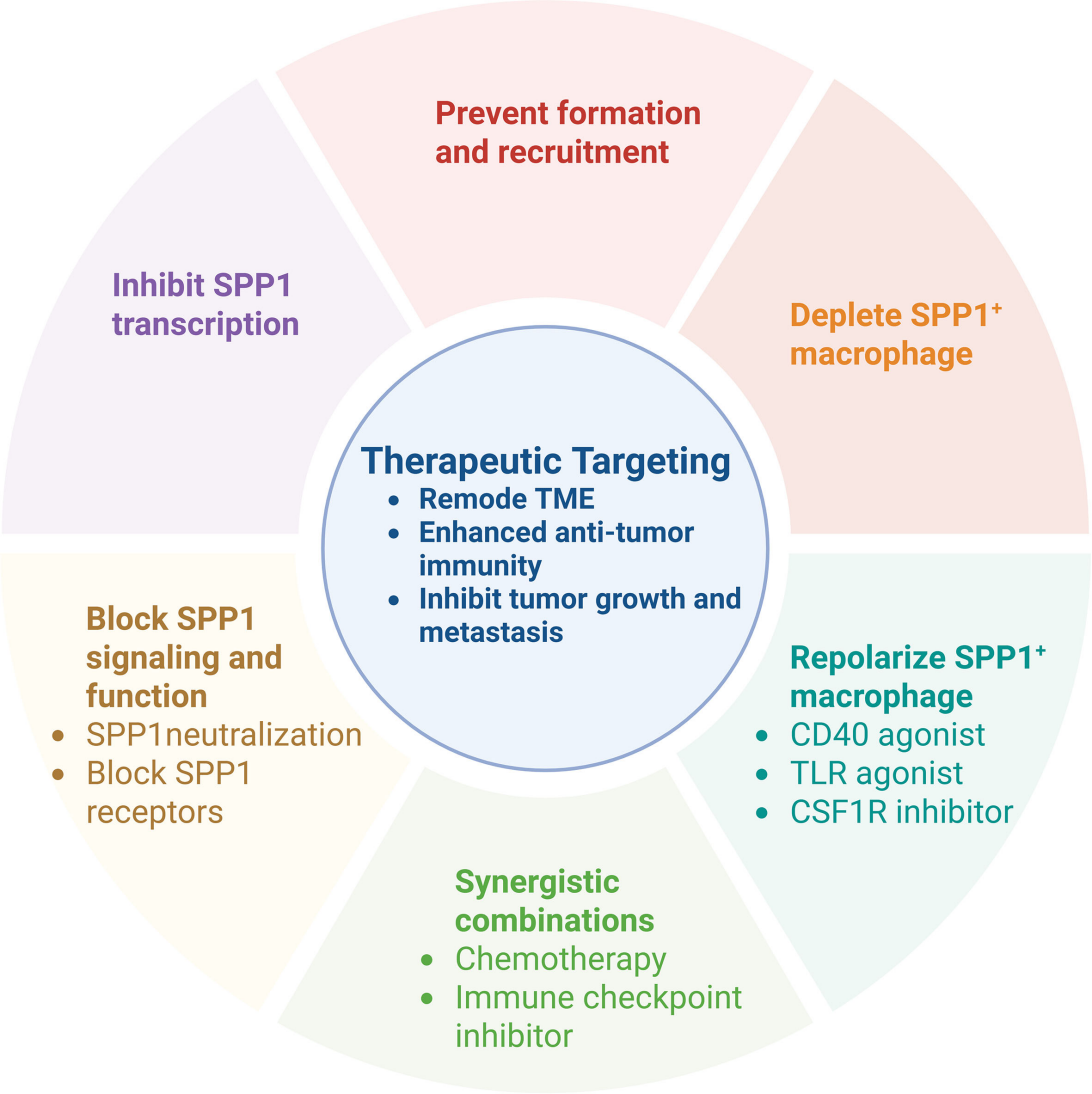
Therapeutic targeting based on SPP1 macrophages. SPP1, secreted phosphoprotein 1; CD, cluster of differentiation; TLR, toll-like receptor; CSF1R, colony stimulating factor 1-receptor.

Targeting SPP1^+^ macrophages represents a promising therapeutic approach currently under investigation. Several strategies have shown potential in preclinical models (**Figure 7**).

### 5.1 Prevention of formation and recruitment of SPP1^+^ macrophages

Blocking monocyte egress from bone marrow and their recruitment into diseased tissue represents a key strategy. This can be achieved through CCR2 inhibitors or CCL2-neutralizing antibodies.

### 5.2 Depletion or repolarization of SPP1^+^ macrophages

Antibody-drug conjugates or CAR-M cells can be employed to selectively eliminate SPP1^+^ macrophages. Alternatively, agonists such as TLR agonists may repolarize these cells from a pro-tumor (M2-like) to an anti-tumor (M1-like) phenotype.

### 5.3 Blocking SPP1 signaling and function

#### 5.3.1 SPP1 neutralization

Monoclonal antibodies that bind SPP1 can prevent its interaction with receptors such as αvβ3 integrin and CD44. Preclinical studies in ovarian cancer models showed that anti-SPP1 antibody treatment reduced tumor volume, accompanied by improved immune cell function.

#### 5.3.2 Blocking SPP1 receptors

Inhibitors or antibodies targeting integrins (e.g., αvβ3) or CD44 can disrupt SPP1 signaling. For instance, OPN-R3 inhibits the binding of OPN to αvβ3 integrin and CD44, suppressing tumor progression and metastasis in breast cancer models (133). Similarly, the anti-OPN mAb AOM1, which specifically blocks integrin αvβ3 binding and thrombin cleavage domains, inhibits tumor invasion and metastasis in lung cancer *in vivo* and *in vitro* (134).

#### 5.3.3 Inhibition of SPP1 transcription

Kinase inhibitors that target upstream signaling pathways can suppress SPP1 production in macrophages (135).

#### 5.3.4 Cytokine pathway inhibition

In head and neck cancer models, targeting SPP1^+^ macrophage derived cytokines, particularly TNF-α and IL-1β with the inhibitor significantly impaired tumor cell proliferation and migration *in vitro* and reduced tumor growth *in vivo*.

#### 5.3.5 Targeting upstream regulators

In aggressive cancers, VSIG4 blockade combined with SPP1 inhibition synergistically enhanced anti-tumor immunity by remodeling the TME and restoring CD8^+^ T cell function.

#### 5.3.6 Disrupting feedback loops

In hepatocellular carcinoma (HCC), targeting the integrin αvβ5 pathway or the CCL15-CCR1 axis may disrupt the reciprocal activation between SPP1^+^ macrophages and cancer stem cells, potentially overcoming chemo-resistance.

### 5.4 Synergistic combinations

SPP1^+^ macrophages contribute significantly to resistance against standard therapies. Targeting these cells can resensitize the TME. Combining SPP1-targeted approaches with immunotherapy such as anti-PD-1/PD-L1 agents can alleviate immunosuppressive barriers, enabling effective T cell-mediated tumor attack.

## 6 Future perspectives and research directions

Studies on SPP1^+^ macrophages is advancing from descriptive characterization toward functional validation and therapeutic targeting. Future efforts should prioritize elucidating the precise mechanisms of SPP1 action, developing specific inhibitors, and designing combination strategies.

### 6.1 Therapeutic targeting of SPP1^+^ macrophages

#### 6.1.1 Inhibitor development

There is a growing need to develop highly selective inhibitors targeting SPP1 or its receptors, such as CD44.

#### 6.1.2 Antibody-based therapies

Anti-SPP1 antibodies have shown promising results in preclinical studies. Future studies should aim to develop humanized antibodies to facilitate clinical application.

#### 6.1.3 Combination therapies

Combining SPP1 blockade with existing immunotherapies (e.g., immune checkpoint inhibitors) may synergistically enhance anti-tumor efficacy.

### 6.2 Mechanistic insights and signaling pathways

#### 6.2.1 SPP1–CD44/integrins axis

Future studies should further investigate downstream signaling events, which induce TEX.

#### 6.2.2 Epigenetic regulation

Emerging evidence indicates that VSIG4+ tumor-associated macrophages (TAMs) promote SPP1 expression through lactate-induced histone H3 lysin 18 lactylation (H3K18la). Exploring additional epigenetic regulators of SPP1 expression may reveal novel therapeutic targets.

#### 6.2.3 Metabolic reprogramming

Understanding how metabolic rewiring such as hypoxia induced changes affects SPP1 expression and function may provide insights for targeting metabolic pathways to modulate immune responses.

### 6.3 Technological innovations and methodologies

#### 6.3.1 Single-cell multi-omics

Integration of single-cell RNA sequencing with spatial transcriptomics (e.g., Stereo-seq) and proteomics (e.g., CODEX imaging) will help delineate the heterogeneity of SPP1^+^ macrophages across diseases and elucidate their spatial interactions with other cells.

#### 6.3.2 Nanoparticle-based delivery

Developing nanoparticle systems for targeted delivery of SPP1 inhibitors or antibodies to tumors could improve therapeutic efficacy and reduce off-target effects.

#### 6.3.3 Humanized mouse models

The use of humanized mouse models that reconstitute the human immune system will be critical for preclinical validation of therapies targeting SPP1^+^ macrophages.

### 6.4 Tumor-specific applications

SPP1^+^ macrophages contribute to immunotherapy resistance in multiple cancers, including prostate, ovarian, and liver cancers. Future research should focus on identifying biomarkers (e.g., serum SPP1 levels) to predict patient responses to SPP1-targeted therapies and investigating the temporal dynamics of SPP1^+^ macrophage accumulation during disease progression to identify optimal windows for therapeutic intervention.

## 7 Conclusion

SPP1^+^ macrophages constitute a functionally distinct and clinically significant subset of tumor-associated macrophages that contribute to cancer progression through multiple mechanisms. By driving immunosuppression primarily via the SPP1–CD44/integrin axis, these cells play a central role in promoting aggressive tumor behavior and are consistently correlated with poor clinical outcomes across various cancer types. The strong association between SPP1^+^ macrophage infiltration and adverse prognosis, supported by compelling preclinical evidence targeting this population, highlights their therapeutic potential. As ongoing research further elucidates the complex biology and interactions of SPP1^+^ macrophages within the tumor microenvironment, they are increasingly regarded as promising biomarkers and therapeutic targets that could improve patient outcomes. The continued development of strategies to target SPP1^+^ macrophages whether through direct SPP1 neutralization, blockade of their pro-tumoral functions, or disruption of their survival signals represents an emerging frontier in cancer therapy. Such approaches may complement existing treatments and help overcome therapeutic resistance in advanced malignancies. Notably, while single-cell RNA sequencing has revealed functional heterogeneity within SPP1^+^ TAMs, this technology remains impractical for routine clinical implementation. Thus, simplified surrogate methodologies such as quantifying SPP1^+^/FAP^+^ cellular co-localization via standardized 3-plex immunohistochemistry protocols can be used to guide therapeutic decisions.
